# Supplementary material for: A Patient with Corticobasal Syndrome and Progressive Non-Fluent Aphasia (CBS-PNFA), with Variants in ATP7B, SETX, SORL1, and FOXP1 Genes
Source: Genes (Basel). 2022 Dec 14;13(12):2361. doi: 10.3390/genes13122361 (PMC9778325; doi:10.3390/genes13122361)
Supplement: Supplementary file 1 [file genes-13-02361-s001.zip › genes-2059640-supplementary/genes-2059640-Supplementary material Clinical assessment proofreading.pdf]

Clinical assessment supplementary

Figure S2. Case timeline

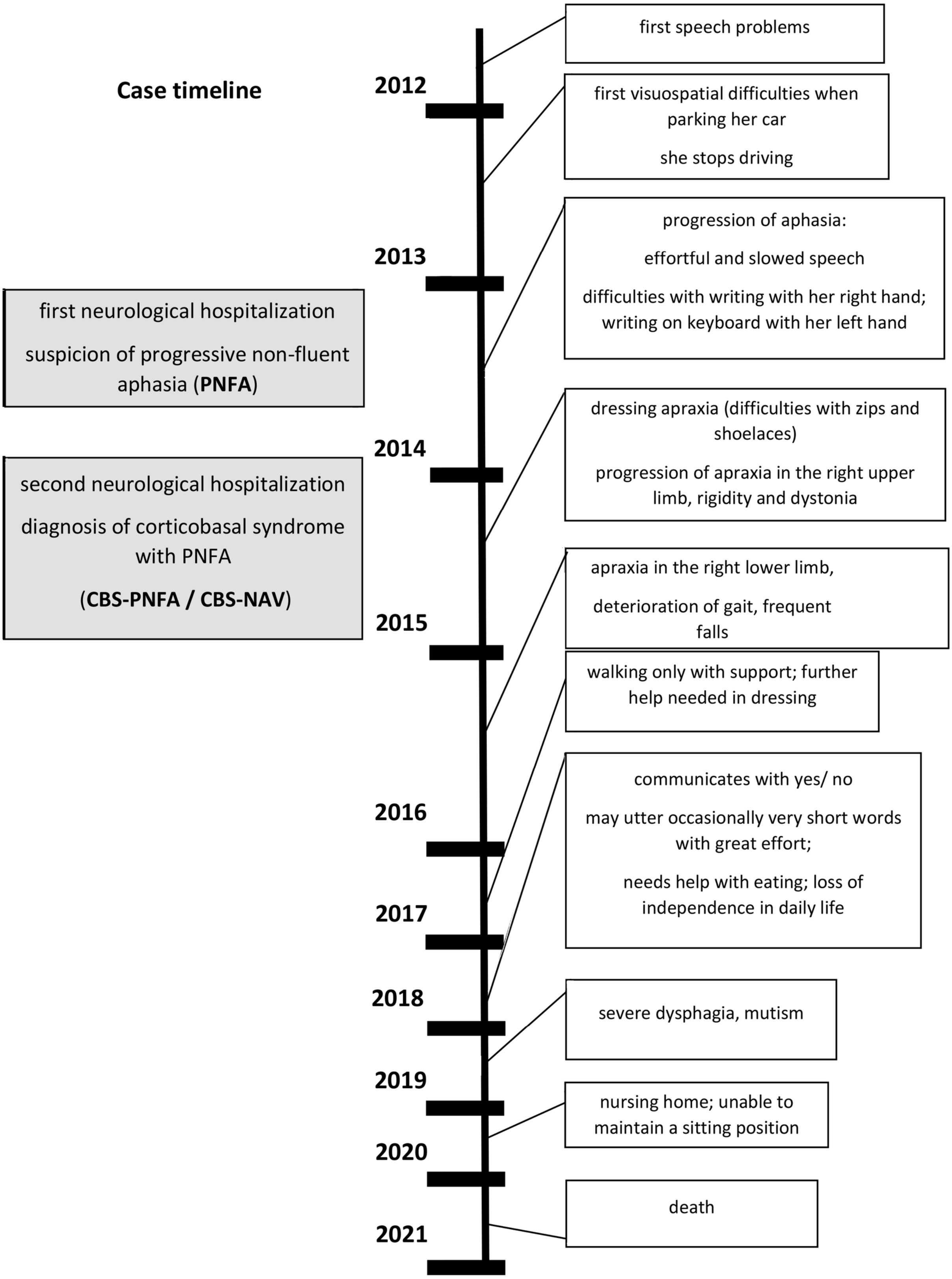

Neuropsychological assessment Table S5. Cognitive and behavioral screening test results in the proband (see also Fig. 1C,D)

	The patient's age – years, months			
	67,2	*68,4*	*69,3*	*70,2**
MMSE / 30	28	25	-	-
ACE-III / 100	-	70	56	-
ACE-III – attention / 18	-	16	12	-
ACE-III – memory / 26	-	19	16	-
ACE-III – fluency / 14	-	1	0	-
ACE-III - language / 26	-	22	20	-
ACE-III – visuospatial / 16	-	12	8	-
FBI – negative behaviour score<br>FBI- disinhibition score<br>FBI – total score (cut-off: 17)	10<br>6<br>16	12<br>3<br>15	-	-

ACE-III, Addenbrooke's Cognitive Examination-III; FBI, Frontal Behavioural Inventory; MMSE, Mini-Mental State Examination

*not feasible due to severe speech apraxia

Neuropsychological assessment Figure S3. Profile of Addenbrooke's Cognitive Examination-III performance in the proband performed twice in the disease course

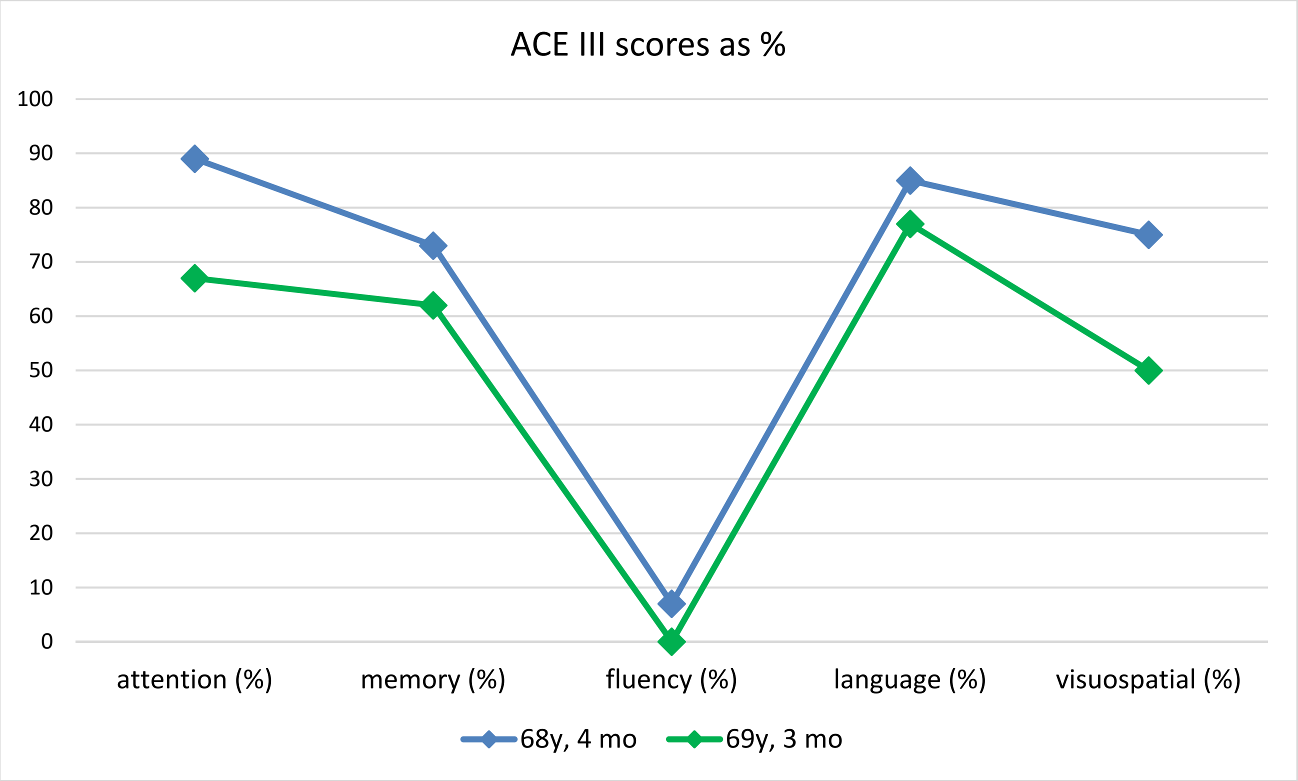

Neuropsychological assessment Table S6. Speech and language evaluation results in the proband

	The patient's age – years, months			
	67,2	*68,4*	*69,3*	*70,2**
Confrontation naming & semantic memory				
BNT / 30	26	20	17	-
SydBAT – Naming / 30	20	20	15	-
SydBAT – Word comprehension / 30	29	26	26	19
SydBAT – Semantic association / 30	21	24	22	19
PALS –naming - animals / 10	10	8	8	-
PALS – word comprehension – animals / 10	10	10	10	-
PALS – naming – objects / 10	9	9	8	-
PALS – word comprehension – objects / 10	10	10	10	10
Verbal fluency				
Semantic fluency – animals / fruit and vegetables	6 / 5	4 / 6	2 / 2	-
Phonemic fluency – K / P / M / S	2 / 4 / 2 / -	2 / 1 / 2 / 2	1 / 1 / 1 / 1	-
Language comprehension				
Commands from BDAE / 15	12	13	11	11
Token Test / 163	138	140	123	118
Understanding Polish grammatical constructions / 10	5	6	5	-
Repetition				
Phrase repetition from BDAE<br>High frequency / low frequency / 8	7 / 8	7 / 5	7/6	-
Word and syllable repetition	slowed repetition rate	impaired complex word repetition	impaired word repetition	impaired syllable repetition
Speech apraxia				
Qualitative assessment	mild	moderate	severe	very

				severe
Oral apraxia	mild	-	-	-
Reading				
Word reading from BDAE / 30	29	29	27	-
Sentence reading from BDAE / 10	10	7	7	-
Writing				
Primer level dictation from BDAE / 15	15	15	15*	-
Writing words to dictation from BDAE / 10	10	10	-	-
Written picture naming / 10	10	10	-	-
Written picture description	agrammatism	agrammatism	-	-
Sentence copy	preserved	preserved	-	-

BDAE, Boston Diagnostic Aphasia Examination; BNT, Boston Naming Test; PALS, Progressive Aphasia Language Scale; SydBAT, Sydney Language Battery

*expressive (both oral and written) language assessment was very limited due to severe speech apraxia and limb apraxia; **written with the left hand

Neuropsychological assessment Table S7. Visuospatial, calculation, memory and executive function evaluation results in the proband

	The patient's age – years, months			
	67,2	*68,4*	*69,3*	*70,2*
Visuospatial function				
VOSP- Incomplete letters / 20	18	17	18	-
VOSP- Dot counting / 10	10	9	8	5
VOSP- Position discrimination/ 20	20	19	18	-
VOSP- Number location/ 10	10	7	3	-
VOSP- Cube analysis / 10	10	8	9	4
CFT copy – Rey / Taylor / 36	34	25	-	-
Benton three-dimensional praxis / 29	-	28	27	-
Line cancellation	-	-	1 right-sided ommission	-
BIT – letter cancellation / 20* - 20**	-	-	14 - 15	-
Bell test / 15*- 15**	-	-	13 - 11	-
Calculation				
Calculation score from WAB (multiple choice format) / 24	24	24	24	22
Serial sevens / 5	4	3	1	-
Memory				
Working memory				-
Months forward / 12; time in sec.	12; 19	12; 26	10; -	
Months backwards / 12; time in sec.	12; 51	11; 69	-	
TMT A	-	233 s.	-	
TMT B	-	756 s.; 6 errors	-	
Verbal learning				-
10-word list learning- curve	3-5-4-7-6	4-5-5-5-4	4-4-4-4-3	

Delayed recall<br><br>Recognition [correct / false positives]	5<br><br>10	4<br><br>10	4<br><br>9	
Visual memory<br><br>selecting previously shown elements from distractors, accuracy in %	100	100	100	100
LLT – displacement score (percentile)<br><br>LLT- delayed recall (percentile)	-<br><br>-	30<br><br>30-85	-<br><br>-	-<br><br>-
CFT – delayed copy / 36	20	21	-	-
Executive function				
Screening- FAB / 18	-	8	8	-
Abstract thinking and cognitive flexibility<br><br>Sorting Weigl blocks / % correct<br><br>The Brixton Spatial Anticipation Test, scaled score	 <br>73<br><br>2	 <br>66<br><br>2	 <br>66<br><br>-	-

BIT, Behavioural Inattention Test; CFT, Complex Figure Test; FAB, Frontal Assessment Batttery; LLT, Location Learning Test; VOSP, Visual Object and Space Perception Test; WAB, Western Aphasia Battery

*left; ** right

Neuropsychological assessment Table S8. Praxis, finger identification, orientation in the body schema, cortical sensory loss and tactile recognition evaluation results in the proband (RUP, right upper limb; RLL, right lower limb)

	The patient's age – years, months			
	67,2	*68,4*	*69,3*	*70,2*
akinesia / hypokinesia	hypokinesia in RUP		hypokinesia in RUL and RLL	akinesia in RUL; hypokinesia in RLL
Synkinesis (hand-foot, hand-mouth)	present	present	present	present
Alien limb symptoms	RUL	RUL	RUL	RUL<br>RLL
Manual dexterity - pegboard tasks				
9 Hole Peg test<br>- right hand (time)<br>- left hand (time)	59 sec.; - 10 SD<br>37 sec. ; - 5 SD	211 sec.<br>54 sec.	impossible<br>93 sec.	impossible<br>259 sec.
Purdue Pegboard<br>- right hand (mean - pegs)<br>- left hand (mean - pegs)<br>- both hands (mean - pegs)	4.66;- 5 SD<br>7.66; - 3.5 SD<br>2; - 4SD	2.67;<br>5.33;<br>0.67;	impossible<br>3.33<br>impossible	-
Praxis				
Alternate hand movements	extinction in the right hand	impossible	impossible	impossible
Spatial praxis<br>Interlocking fingers test / 4	2	3	0	impossible
Ideomotor praxis	impaired mostly in the right hand		moderately impaired also in the left hand	severely impaired
Efferent motor praxis (left hand) / 5	1	0	-	-
Lower limb praxis	preserved	impaired in the RLL		impaired in both lower

				limbs
Benton body schema orientation / 20	-	17	7	-
Left-right orientation<br>Manikin test / 32	-	-	18	-
Cortical sensory loss				
Tactile double stimulation	-	-	no sensory loss, no extinction	-
Recognition of 4 shapes written in a palm<br>- right / 8<br>- left / 8	6<br>8	8<br>8	7<br>8	-
Finger recognition<br>- right hand (with / without visual control) / 10<br>- left hand (with / without visual control) / 10	10 / 9<br>10 / 10	10 / 10<br>10 / 10	8 / 7<br>10 / 10	-
Tactile object recognition<br>- right hand / 10<br>- left hand / 10	-<br>-	9<br>10	6<br>8	-

Table S10. Clinical phenotypes, biochemical and brain abnormalities observed in carriers of single heterozygous *ATP7B* mutation (Hzgs)

No	Diagnosis/ Clinical phenotype/follow up time	Brain imaging abnormalities (suggestive of free copper accumulation)	AAO	Cognitive function	Biochemical analyses	*ATP7B* Mutation	reference
1	Asymptomatic*	n.a.	-	n.a.	serum ceruloplasmin decreased in 3 out of 12 Hzc; serum copper decreased in 1 out of 12 Hzc		[1]
2	Asymptomatic*	n.a.	-	n.a.	serum ceruloplasmin decreased in 16 out of 16 Hzc		[2]
3	Asymptomatic*	changes in MRS in both the pallidum and thalami compared to control subjects	-	n.a.	n.a.		[1]
4	Asymptomatic*	more frequent bilateral lenticular nucleus and substantia nigra hyperechogenicity than in control individuals	-	n.a.	n.a.		[3]
5	EOPD; Very slowly progressing levodopa-responsive hemiparkinsonism and dysarthria/7 years	n.a.	39	preserved in MMSE**, CDT*** , FAB****	lower serum ceruloplasmin; elevated serum nonceruloplasmin copper	C1079G/exon 14 SIFT:0.05; Poly-Phen2: 0.999; CADD:32	[4]
6	LOPD; major depression and parkinsonism (three sisters)	n.a.	67, 69, 70	preserved in MMSE	n.a.	(-441/-427 del) 15 bp deletion in the 5' UTR region	[5]
7	EOPD; 65-year-old patient	n.a.	50		n.a.	H1069Q,	[6]

	presented advanced PD with end-of-dose akinesia and intermittent L-dopa–induced dyskinesias under a predominantly dopaminergic therapy.					this mutation was also present in asymptomatic 52-year-old sister of the patient	
8	Wilson's disease; limb weakness and tremor, after 1 year treatment of penicillamine (metal chelator) weakness of the upper limbs and tremor were relieved.	n.a.	30		lower serum ceruloplasmin	R827Q/exon 10	[7]
9	Our patient: CBS=NAV	cortical atrophy of the both frontal lobes, T1- and T2-weighted MRI images didn't reveal any hyper- and hypointensities in the basal ganglia. MRS and transcranial US were not performed.	63	corticobasal syndrome, non-fluent aphasia, apraxia of speech, right-sided hemi-spatial neglect, limb apraxia, executive dysfunction	n.a.	H1069Q/exon 14	this manuscript

AAO – age at onset

*- carriers of single heterozygous *ATP7B* mutation (Hzcs) were recruited from WD patients' families

**MMSE - Mini-Mental State Examination

*** - Clock Drawing Test

****- Frontal Assessment Battery

MRS- Magnetic resonance spectroscopy ;

Only in case no 5, the clinical symptoms of EOPD were accompanied by typical biochemical hallmarks of the Wilson's disease (Ilyechova et al. 2019) speaking in favor of the causative role of exon 14 ATP7B mutation.

References:

1. Tarnacka, B.; Szeszkowski, W.; Buettner, J.; Golebiowski, M.; Gromadzka, G.; Czlonkowska, A. Heterozygous carriers for Wilson's disease--magnetic spectroscopy changes in the brain. *Metab Brain Dis* **2009**, *24*, 463-468,
2. Manoochehri, J.; Masoumi Dehshiri, R.; Faraji, H.; Mohammadi, S.; Dastsooz, H.; Moradi, T.; Rezaei, E.; Sadeghi, K.; Fardaei, M. Family screening for a novel ATP7B gene mutation, c.2335T>G, in the South of Iran. *Iran J Ped Hematol Oncol* **2014**, *4*, 26-31,
3. Skowronska, M.; Litwin, T.; Kurkowska-Jastrzebska, I.; Czlonkowska, A. Transcranial sonography changes in heterozygotic carriers of the ATP7B gene. *Neurol Sci* **2020**, *41*, 2605-2612,
4. Ilyechova, E.Y.; Miliukhina, I.V.; Karpenko, M.N.; Orlov, I.A.; Puchkova, L.V.; Samsonov, S.A. Case of Early-Onset Parkinson's Disease in a Heterozygous Mutation Carrier of the ATP7B Gene. *J Pers Med* **2019**, *9*,
5. Sechi, G.; Antonio Cocco, G.; Errigo, A.; Deiana, L.; Rosati, G.; Agnetti, V.; Stephen Paulus, K.; Mario Pes, G. Three sisters with very-late-onset major depression and parkinsonism. *Parkinsonism Relat Disord* **2007**, *13*, 122-125,
6. Moller, J.C.; Leinweber, B.; Rissling, I.; Oertel, W.H.; Bandmann, O.; Schmidt, H.H. Prevalence of the H1069Q mutation in ATP7B in discordant pairs with early-onset Parkinson's disease. *Mov Disord* **2006**, *21*, 1789-1790,
7. Zhang, Z.; Liu, J.; Zheng, W.; Hou, Q.; Zhang, L. A novel heterozygous carrier of ATP7B mutation with muscle weakness and tremor: A Chinese Case Report. *J Musculoskelet Neuronal Interact* **2020**, *20*, 614-618,
