# Supplementary material for: A Patient with Corticobasal Syndrome and Progressive Non-Fluent Aphasia (CBS-PNFA), with Variants in ATP7B, SETX, SORL1, and FOXP1 Genes
Source: Genes (Basel). 2022 Dec 14;13(12):2361. doi: 10.3390/genes13122361 (PMC9778325; doi:10.3390/genes13122361)

Supplementary material genetics

Figure S1. Sanger sequencing confirming the presence of *ATP7B* c.3207C>A variant (primer ATP7BEx13F).

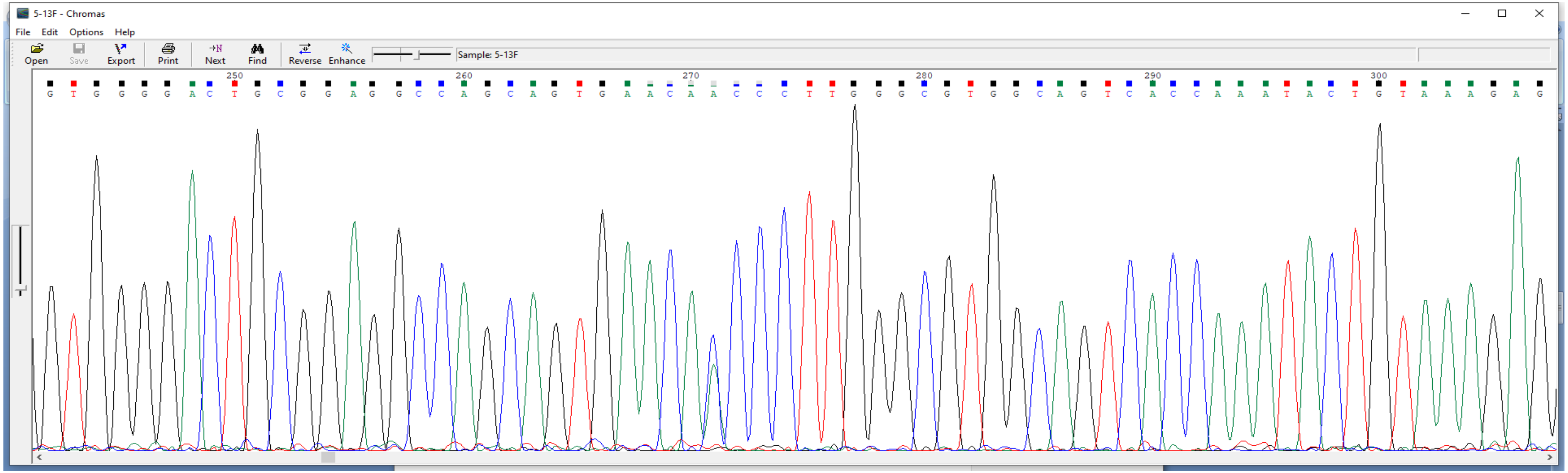

Table S4. Primers used in the study

Gene/application	Primer name	sequence	reference
ATP7B (Sanger sequencing)	ATP7BEx13F<br>ATP7BEx15R	TTTCCAGACGTCCATCACGG<br>GGAAGTCCGTGCAGTATCCC	Designed for this study (NCBI primer blast)
GAPDH	GAPDHF<br>GAPDHR	GTTCGACAGTCAGCCGCATC<br>GGAATTTGCCATGGGTGGA	[1]
PPIB	PPIBF<br>PPIBR	GGTGATCTTTGGTCTCTTCGG<br>TAGATGCTCTTTCCTCCTGTG	[2]
DSCR1	DSCR1F<br>DSCR1R	GCACCAGCTCAAGAAGGAACC<br>GGGACTCAAATTTGGCCCTGG	[3]
SETX (Real time PCR analysis)	SETXRT10F<br>SETXRT10R	GGTCCAGAAAGGGGATGTGA<br>CGTTTTGAGGTTTTAGCAAGAGC<br>primers are color highlighted in Scheme 1	Designed for this study (NCBI primer blast)
SETX (isoform analysis: PCR and Sanger sequencing)	SETX10F<br>SETX10R	GGTCCAGAAAGGGGATGTGA<br>GTCAGGGGCCTGTTCTCTTG<br>primers are color highlighted in Scheme 1	Designed for this study (NCBI primer blast)

References:

1. Chung, S.W.; Choi, B.M.; Kim, J.Y.; Lee, Y.S.; Yoon, J.P.; Oh, K.S.; Park, K.S. Altered Gene and Protein Expressions in Torn Rotator Cuff Tendon Tissues in Diabetic Patients. *Arthroscopy* **2017**, *33*, 518-526 e511,
2. Lefever, S.; Vandesompele, J.; Speleman, F.; Pattyn, F. RTPrimerDB: the portal for real-time PCR primers and probes. *Nucleic Acids Res* **2009**, *37*, D942-945,
3. Gong, C.; Kim, Y.K.; Woeller, C.F.; Tang, Y.; Maquat, L.E. SMD and NMD are competitive pathways that contribute to myogenesis: effects on PAX3 and myogenin mRNAs. *Genes Dev* **2009**, *23*, 54-66,

Table S9. Human Splicing Finder analysis of SETX variant (rs755971927, c.2385_2387delAAA, p.Ile795_Lys796delinsMe) detected in the patient.

HSF analysis verdict: new donor splice site, activation of a cryptic donor site. Potential alteration of splicing.

	name	position	sequences	variation
1 (highlighted in magenta color) in Scheme S1	HSF Donor site (matrix GT)	chr9:132329218	GTAATAAAG>CATGTAATA	43.74>77.11 (76.29%)
2 (highlighted in sea green) in Scheme S1	HSF Donor site (matrix GT)	chr9:132329228	GTTATCACA>AAAGTTATC	36.57>70.95 (94.01%)

Scheme S1. The scheme of aberrant splicing in the patient’s fibroblasts carrying variant *SETX* c.2385_2387delAAA (rs755971927) in exon 10 - deletion of AAA (highlighted in red)

SETX Exon 10 sequence (Transcript: ENST00000224140.6; ensembl)

GATTCTGGATGGAGAACAGCCATTTGCCCAGATTATTGTCCTAACATGTATGAAGAAATG

GAAACATTAGCCAGTGTACTTCAGTCAGATATTGGTCAAGACATGCGTGTTCATAACAGC

ACATTTCTATGGTTCATCCCTTTTGTCCAGTCCCTCATGGATCTTAAGGATTTGGGTGTG

GCTTACATAGCACAGGTTGTTAATCATCTGTACTCTGAAGTCAAAGAAGTCCTCAACCAA

ACAGATGCTGTGTGTGACAAAGTCACTGAATTTTTTCTTCTAATTTTGGTATCAGTGATT

GAACTGCATAGAAATAAAAAATGTTTGCATTTGCTGTGGGTAAGTTCCCAGCAATGGGTG

GAAGCCGTCGTCAAATGTGCCAAGCTTCCTACCACTGCGTTTACACGGAGTTCTGAGAAA

TCATCTGGAAATTGCTCCAAAGGAACAGCAATGATATCTTCACTGTCATTGCATTCCATG

CCATCTAACTCTGTACAACTTGCTTATGTGCAGCTGATTAGAAGTCTCCTTAAAGAAGGT

TATCAGCTTGGGCAGCAGTCTCTTTGCAAGCGATTCTGGGATAAGCTCAACTTATTCCTT

AGAGGAAATTTATCTCTAGGTTGGCAGTTGACTAGTCAGGAAACCCATGAGCTACAAAGT

TGCTTAAAGCAAATTATTAGAAACATAAAATTCAAAGCACCTCCATGTAACACTTTTGTG

GATCTGACTTCTGCATGTAAAATCTCTCCTGCATCTTATAATAAAGAAGAAAGTGAACAA

ATGGGGAAGACGTCTAGAAAAGATATGCATTGTTTGGAAGCTTCCAGCCCAACATTTTCT

AAAGAACCAATGAAAGTGCAAGACAGTGTATTGATCAAAGCAGATAACACTATAGAAGGT

GACAATAATGAGCAAAATTATATAAAGGATGTGAAACTAGAGGACCATCTCTTAGCTGGG

TCATGCTTAAAGCAGAGTAGTAAAAACATTTTTACTGAAAGAGCTGAAGATCAAATTAAA

ATAAGTACAAGGAAGCAGAAGTCTGTAAAAGAGATCTCTTCATATACACCAAAGGACTGT

ACTTCAAGAAATGGTCCAGAAAGGGGATGTGACAGAGGAATAATAGTATCAACACGTTTG

TTGACTGATTCTAGCACTGATGCTTTGGAAAAAGTGTCCACATCGAATGAAGATTTCTCT

TTAAAGGATGATGCTCTTGCTAAAACCTCAAAACGAAAAACTAAG**GT**ACAGAAAGATGAA

ATCTGTGCAAAGTTATCACATGTAATAAAGAAGCAACACAGGAAGAGTACTTTGGTCGAT

AATACTATCAATTTAGATGAAAATTTGACTGTATCTAACATTGAGAGTTTCTATTCAAGG

AAAGATACAGGAGTTCAGAAAGGAGATGGTTTCATACACAATCTTTCTTTAGACCCTAGT

GGTGTTCTGGATGATAAGAATGGAGAACAAAAATCTCAAAACAATGTATTGCCAAAAGAG

AAACAATTAAAGAATGAAGAATTAGTTATTTTCTCTTTCCATGAAAACAATTGTAAAATA

CAGGAATTTCATGTTGATGGTAAAGAATTGATCCCTTTTAC**AG**AAATGACCAATGCTTCA

GAGAAGAAATCATCTCCCTTTAAAGATCTTATGACTGTACCTGAATCAAGAGATGAGGAG

ATGAGTAATAGTACCAGTGTGATTTATTCTAACTTGACAAGAGAACAGGCCCCTGACATC

AGTCCTAAATCTGACACCTTAACGGATTCTCAGATAGACAGAGACCTTCACAAATTATCT

TTACTAGCTCAAGCCAGTGTTATTACGTTCCCATCCGATTCACCTCAGAACTCATCGCAG

CTGCAAAGGAAAGTAAAAGAAGATAAAAGATGTTTCACAGCTAACCAAAATAATGTTGGA

GATACCTCCCGTGGACAGGTTATTATTATTTCAGATTCTGATGATGATGATGATGAAAGA

ATCCTGAGTCTTGAGAAACTCACTAAACAGGACAAAATATGCCTTGAGAGGGAACATCCA

GAGCAGCACGTTTCAACAGTTAATAGTAAGGAGGAAAAGAATCCAGTAAAGGAAGAAAAG

ACAGAGACTCTTTTTCAGTTTGAGGAATCTGATTCTCAGTGTTTTGAGTTTGAAAGTTCA

TCTGAAGTGTTTTCAGTTTGGCAAGATCATCCAGACGATAATAATTCAGTTCAAGATGGT

GAGAAAAAATGTTTGGCTCCTATAGCCAATACTACAAATGGTCAGGGTTGTACAGATTAT

GTATCTGAAGTTGTTAAAAAAGGAGCAGAGGGCATTGAAGAACACACAAGACCACGGAGT

ATTTCTGTTGAAGAATTTTGTGAAATTGAAGTAAAAAAGCCTAAGAGAAAACGATCTGAA

AAACCAATGGCTGAAGATCCTGTGAGGCCTTCATCTTCTGTCAGAAATGAGGGCCAGTCT

GATACTAATAAGAGAGATCTTGTGGGAAATGATTTTAAAAGTATTGATAGAAGGACTTCA

ACTCCCAATTCACGTATTCAGAGAGCCACTACGGTTTCACAAAAGAAGTCTTCAAAGCTT

TGTACTTGTACAGAACCCATCAGGAAAGTTCCAGTTTCTAAGACCCCTAAGAAAACTCAT

TCAGATGCCAAAAAAGGACAGAATAGAAGTTCAAATTACCTAAGTTGTAGAACAACTCCT

GCTATAGTGCCGCCAAAGAAATTTCGTCAGTGTCCTGAGCCAACTTCAACAGCTGAGAAA

CTTGGCCTGAAAAAGGGTCCTCGTAAGGCATATGAGTTGTCCCAGCGGTCTTTGGATTAT

GTAGCTCAATTACGTGATCATGGCAAAACTGTTGGAGTAGTTGATACCCGAAAAAAGACT

AAATTAATTTCTCCTCAGAACCTGTCTGTCAGAAATAATAAGAAACTTCTGACTAGTCAA

GAACTTCAGATGCAAAGGCAGATCAGACCCAAATCACAAAAAAATAGACGAAGACTTTCT

GATTGTGAAAGTACAGATGTTAAAAGAGCAGGGTCACATACAGCACAGAATTCTGACATA

TTTGTACCAGAATCTGATAGGTCAGATTATAATTGTACAGGAGGAACTGAGGTACTTGCC

AACAGTAACAGAAAACAGTTAATAAAATGCATGCCTTCTGAACCAGAAACCATAAAAGCA

AAACATGGGTCTCCAGCAACTGATGATGCTTGCCCTTTGAACCAGTGTGATTCTGTAGTG

TTAAATGGAACAGTACCAACAAATGAAGTAATTGTCTCCACTTCAGAAGACCCTCTGGGT

GGAGGTGATCCAACAGCACGTCATATAGAGATGGCAGCTTTGAAAGAAGGAGAGCCTGAC

TCCAGCAGTGATGCAGAGGAAGATAACTTATTTTTAACCCAAAATGATCCTGAAGATATG

GATTTATGTTCACAAATGGAGAATGACAATTATAAACTCATTGAACTAATTCATGGAAAA

GATACAGTTGAGGTTGAAGAAGATTCTGTAAGTCGGCCTCAGTTGGAATCTTTGAGTGGC

ACAAAGTGTAAGTACAAAGATTGTCTTGAAACCACAAAAAACCAGGGTGAATACTGCCCA

AAACACTCTGAAGTGAAAGCAGCAGATGAAGATGTATTTCGTAAACCTGGCTTGCCTCCT

CCTGCATCTAAACCTTTGAGACCTACCACTAAGATTTTTAGCTCAAAGAGTACTTCACGA

ATTGCTGGTCTTTCTAAATCTTTGGAAACTTCTTCAGCACTTTCACCGTCTCTAAAAAAT

AAGTCAAAGGGGATACAGTCGATTTTGAAAGTACCACAGCCAGTTCCCCTCATAGCTCAG

AAGCCAGTTGGTGAAATGAAGAATTCGTGCAATGTTCTTCATCCTCAGTCTCCGAATAAT

TCCAACAGGCAAGGTTGCAAAGTTCCATTTGGTGAAAGCAAATATTTTCCATCTTCCTCT

CCAGTAAACATTCTTTTGTCATCACAGTCTGTCTCTGACACCTTCGTTAAAGAGGTCTTA

AAATGGAAATATGAAATGTTTTTGAACTTTGGTCAGTGTGGGCCCCCTGCAAGTCTTTGT

CAGTCCATCTCAAGACCTGTGCCTGTCAGATTTCACAATTATGGAGATTATTTTAATGTT

TTTTTCCCTTTGATGGTATTGAATACTTTTGAAACA

The Underlined sequence in SETX Exon 10 sequence above is deleted (358 bp) in isoform A (compare with Figure 2 A and C in the manuscript).

The potential new splice sites determined by Human Splicing Finder analysis are highlighted in magenta and sea green colors in SETX Exon 10 sequence above.

Sequences highlighted in yellow and grey in SETX Exon 10 sequence above correspond to the fragments that remain after 358 bp deletion in isoform A.

>2F.ab1. Sequencing result of band *A excised from agarose gel (compare with Figure 1 line 6, in the manuscript)

GTATCAACACGTTTGTTGACTGATTCTAGCACTGATGCTTTGGAAAAAGTGTCCACATCGAATGAAGATTTCTCTTTAAAGGATGATGCTCTTGCTAAAACCTCAAAACGAAAAA
CTAAGAAATGACCAATGCTTCAGAGAAGAAATCATCTCCCTTTAAAGATCTTATGACTGTACCTGAATCAAGAGATGAGGAGATGAGTAATAGTACCAGTGTGATTTATTCTAA
CTTGACAAGAGAACAGGCCCCTGACA

Figure S4. Sequencing result of band *A excised from agarose gel (compare with Figure 1 line 6)

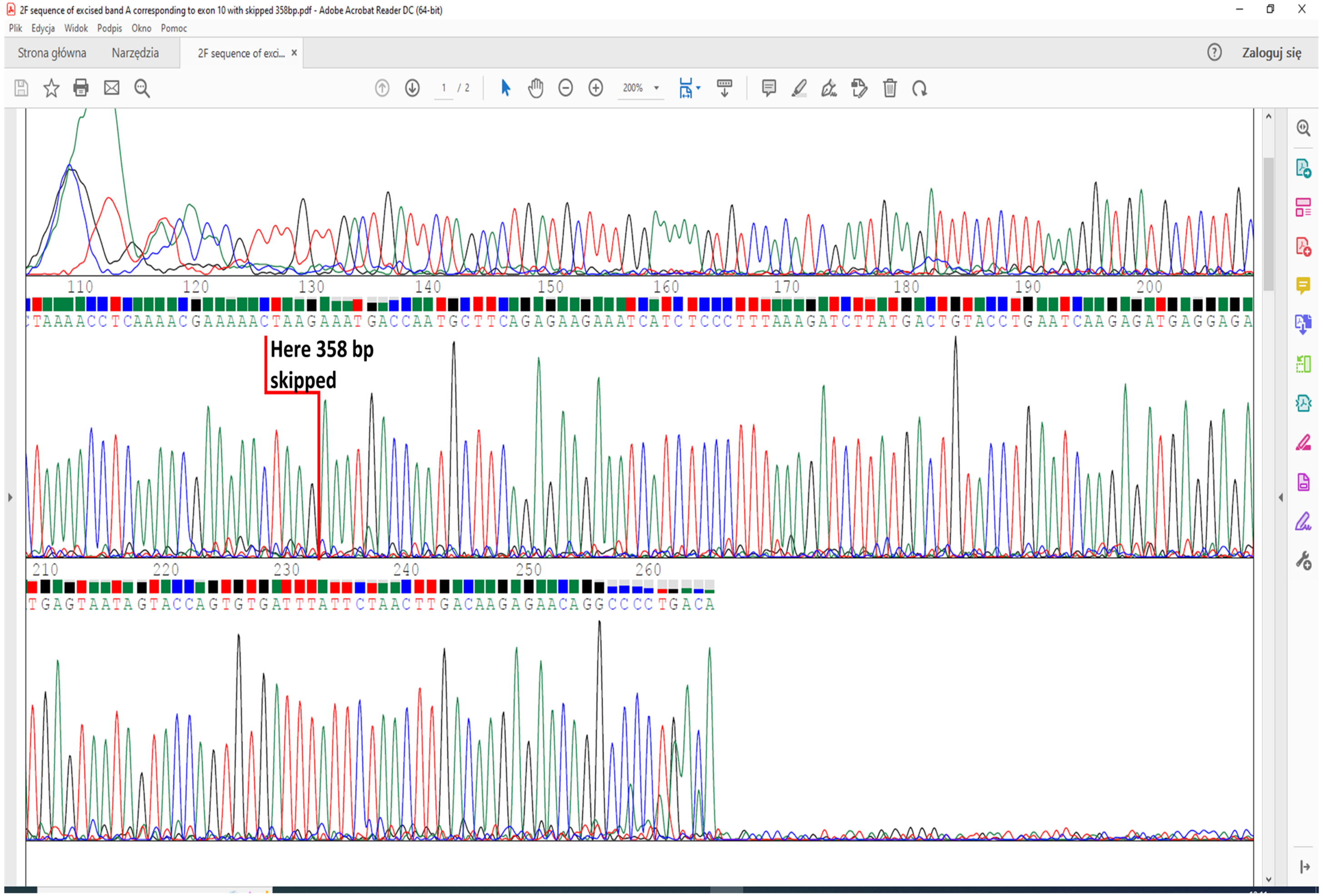

Figure S5. Sequencing result of the main band (see Figure 1 line 6, the strongest band) excised from agarose gel confirmed the presence of *SETX* c.2385_2387delAAA deletion.

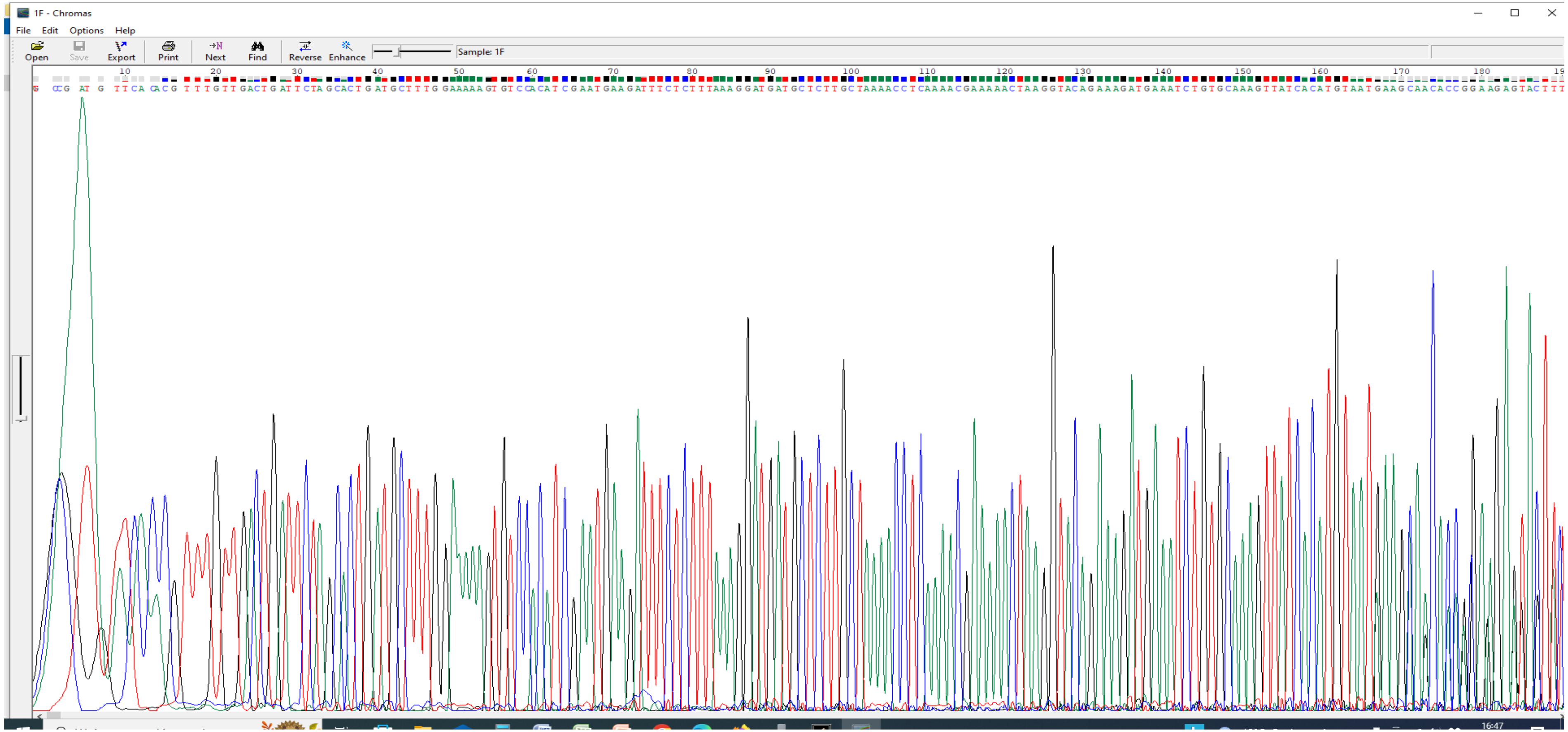

Supplement: Supplementary file 1 [file genes-13-02361-s001.zip › genes-2059640-supplementary/genes-2059640-Supplementary material Genetics proofreading.pdf]
